# Diagnostic criteria patterns of U.S. children with Metabolic Syndrome: NHANES 1999–2002

**DOI:** 10.1186/1475-2891-6-38

**Published:** 2007-11-06

**Authors:** Sibylle Kranz, Lindsey J Mahood, David A Wagstaff

**Affiliations:** 1 Department of Nutritional Sciences, The Pennsylvania State University, 5 Henderson Building, University Park, USA, PA 16802; 2 Health and Human Development (HHD) Consulting Group, The Pennsylvania State University, S153 Henderson Building, University Park, USA, PA 16802

## Abstract

**Background:**

As childhood obesity increases in the U.S., the Metabolic Syndrome (MS) can be assumed to be increasing in the pediatric population as well. To date, there is lack of information on the most prevalent risk factors of MS in children and the patterns of risk factors present in children met the criteria for MS.

**Methods:**

Anthropometric and medical data of children 2–18 years old of a nationally representative data set (NHANES 1999–2002) were obtained and the diagnostic criteria of Cook et al. employed to determine MS prevalence. Three samples were examined: a) Children 2–18 years old with non-missing data on at least three of the five diagnostic criteria but missing blood glucose data (n = 5,172), b) a subsample of 12–18 year olds also providing fasting glucose data but who were not overweight or obese using the International Obesity Task Force (IOTF) standards (n = 1,064), and c) 12–18 year olds with blood glucose data who were overweight or obese (n = 641).

**Results:**

Disease prevalence estimates were 2%, 0.7%, and 23% in the three populations. More than 10% of the children providing fasting blood levels had hyperglycemia. 2% of the overweight or obese 12–18 year olds with fasting blood glucose data met all five diagnostic criteria for MS. In all groups, elevated total triglycerides but low high density lipoprotein (HDL) level affected a large proportion of the population.

**Conclusion:**

Results indicate a reason for concern, since the prevalence of MS risk factors in children was high. Dyslipidemia (concurrent high total triglyceride levels and low HDL levels) were prevalent in large portions of the population, even in the non-overweight. Thus, chronic disease prevention efforts in the pediatric population should not only encourage healthy body weight but also include dietary recommendations to consume diets moderately low in fat with emphasis on polyunsaturated and monounsaturated fats within recommended ratios of omega-6 and omega-3 fatty acids.

## Background

As childhood obesity increases in the United States, the metabolic syndrome (MS) might be rising as well [1]. MS is defined as the concurrence of obesity, impaired glucose metabolism, dyslipidemia, and elevated blood pressure, a grouping of metabolic risk factors that increases the risk for coronary heart disease, atherogenic diseases, and diabetes type 2 [2]. In addition to the disease burden attributable to each individual component of MS, their combination has been found to be independently associated with the development of cardiovascular disease and mortality from all causes in adults [3]. Overweight or obese children and adolescents are at increased risk for some or all of the individual factors of MS [4,5].

In 2003, Cook et al. modified the National Cholesterol Education Panel's (NCEP) diagnostic criteria for adults to establish cut-points relative to the distribution of measurements within the pediatric population using data from the National Health and Nutrition Examination Survey (NHANES) 1988–1994 [6]. To diagnose MS, chronic disease indicators are examined independently of one another. Atherogenic dyslipidemia is determined by assessing total blood triglyceride and high density lipoprotein (HDL) levels, hypertension is defined as increased systolic or diastolic blood pressure, insulin resistance is the presence of elevated fasting blood glucose levels and abdominal obesity is assessed using the child's waist circumference measurements in relation to a reference population. Childhood obesity can also be determined with the ratio of body weight to body height (body mass index (BMI)) and the International Obesity Task Force (IOTF) standards to classify children by age- and gender-specific body weight status cut points [7].

Although there is agreement on the diagnostic criteria for MS in adults, there is no consensus on the appropriate cut-points for diagnosis in children [8,9]. However, there is agreement that a measured fasting blood glucose value of ≥ 100 mg/dL reflects hyperglycemia [10].

The risk factors for MS appear to track from childhood into adulthood [11,12]. Reassuringly, the absence of MS risk factors during childhood has been found to be predictive of lower cardiovascular disease risk in adulthood [13]. Thus, prevention of MS during childhood might not only decrease chronic disease burden early in life but also lower the proportion of adults who will develop the disease.

The aim of this study was to contribute to the understanding of MS risk factors during childhood by examining the diagnostic patterns of MS in nationally representative samples of 2–18 year old children.

## Methods

### Data

The Centers for Disease Control and Prevention (CDC) conducts the National Health and Nutrition Examination Survey (NHANES), an ongoing survey using a multistage, stratified area design to obtain a sample of respondents that is representative of the civilian non-institutionalized U.S. population. A detailed description can be found elsewhere [14]

Levels of blood glucose were ascertained after venal blood draw in the mobile examination vehicle. Fasting blood samples were collected from adolescents 12–18 years old (n = 1,705). Total triglycerides, high density lipoprotein (HDL), and fasting blood glucose levels were provided in milligrams per deciliter (mg/dL).

Blood pressure measurements were obtained by using repeated standard medical procedures, in that systolic and diastolic blood pressure measurements were taken twice. One average value for the systolic as well as the diastolic measurement was calculated and provided as systolic and diastolic blood pressure in millimeters of mercury (mmHg).

Measured height, weight, and waist circumference as well as calculated body mass index (BMI) are available in the NHANES data set. Weight (kilograms) was obtained as the individual stood on a digital scale. Standing height (meters) was measured with an electronic stadiometer in individuals who were at least two years old and waist circumference was measured in centimeters (cm) using a tape. BMI was calculated by dividing body weight (in kilograms) by height (in meters square).

We employed the International Obesity Task force (IOTF) standards to determine children's age- and gender-specific body weight status [7] and created three categories: healthy weight, overweight, and obese. The IOTF standards for overweight and obesity were developed in 2000 by the International Obesity Task Force and are similar to the centile curves of the Centers for Disease Control and Prevention (CDC). However, while the CDC age- and sex specific BMI-for-Age charts are based on a U.S. reference population, IOTF standards were based on the combination of large, nationally representative data sets from six countries (Brazil, Great Britain, Hong Kong, the Netherlands, Singapore, and the United States). Thus, national differences in fatness were considered in this standard. Sex-specific centile curves for BMI were constructed for each data set using the LMS method [15] and superimposed on each other. The resulting cluster of centile curves was positioned to go through the adult cut points for overweight and obesity at age 18 (BMI of 25 and 30, respectively). As a result, international BMI-for-Age cut off points for overweight and obesity based on a heterogeneous, worldwide referent population were generated.

To describe the proportion of children with increased body weight, the CDC's BMI-for-age and gender-specific growth charts were used to classify children as "healthy weight" if their calculated z-score placed them below the 85^th ^percentile, "at risk for overweight" between the 85^th ^and 94^th ^percentile, or "overweight" if it was above the 94^th ^percentile [15].

### Sample Subpopulations

The data set provides dietary, anthropometric, and medical data for 7,672 children. To provide prevalence estimates for the risk factors of MS, children were only included in this analysis if they had non-missing values for at least three of the five diagnostic criteria (n = 5,172) while children with missing data for two or more criteria were excluded from all analysis in this study. To afford a fair comparison of disease risk within the study population, which was from a large range of ages (2–18 years old) and also included children with missing information on one of the five diagnostic criteria for MS (hyperglycemia) three subgroups of the population were created: a) all children 2–18 years old who had non-missing data for at least three diagnostic criteria but did not provide fasting glucose levels (n = 3,467), b) 12–18 year olds who had non-missing data for at least three diagnostic criteria and provided fasting blood glucose data but were not overweight or obese using the IOTF cut points (n = 1,064), and c) a subset of the latter sample that was also classified to be overweight or obese (n = 641). This grouping of the study population allowed the estimation of the proportion of individuals with disease risk by comparing the number of children who met the diagnostic criteria of the MS risk factors to the children who could have met the criteria but whose medical data indicated a healthy condition. For instance, none of the children ages 2–12 years old could have been diagnosed with hyperglycemia due to the lack of fasting glucose measurements. Thus, the prevalence estimate for hyperglycemia was limited to the sample population with blood glucose measurement values in the denominator.

### Disease risk measures

To determine the presence of MS risk factors, measures employed in this study were compared to the cut-points established by Cook et al [6]. Children were considered to have excessive total triglyceride levels if blood concentrations were ≥ 110 mg/dL. High density lipoprotein (HDL) levels were considered low at a level of ≤ 40 mg/dL while fasting blood glucose levels ≥ 100 md/dL were considered indicative of hyperglycemia.

Abdominal obesity was assessed by comparing children's waist circumference measurements to the age- and gender-specific population distribution [16]. Children were considered as having abdominal obesity if they met or exceeded the 90^th ^percentile for age and gender. Hypertension was considered as having an average systolic or diastolic blood pressure of > 90^th ^percentile by age, gender, and ethnic group [17].

A dichotomous variable was created for each one of the five criteria. Children were coded as "= 1" when they met or exceeded Cook's cut-point value for the criteria and "= 0" if not.

To obtain overall prevalence estimates of MS in the U.S. pediatric population, children were classified as having MS when they met any three of the five possible diagnostic criteria in each of the subpopulations.

### Statistical Analysis

Although NHANES data are released in 2-year increments, survey waves were designed to be merged [14] and we combined data from 1999 to 2002. All analysis was weighted and sample design corrected using survey commands in STATA 9.2 to maintain the nationally representative character of the data. As specified by the guidelines for NHANES data analysis, we used individual's 4-year medical weights. Stata's complex sample survey routines (version 9.2; StataCorp LP, College Station, TX[18]) were used to calculate descriptive statistics and the proportion of children meeting the diagnostic criteria for MS. String variables were created to indicate the patterns of 0–1 coding for the five diagnostic criteria in the population of children with non-missing data on at least three of the five criteria.

## Results

### Sample description

More than five thousand children provided measured data on at least three of the five diagnostic criteria for the metabolic syndrome. In all sample groups, approximately half of the children were girls.

The majority of the sample was non-Hispanic white. Of the teenagers 12–18 years old, 1,705 children provided data on fasting blood glucose levels. Of those, 1,064 children were not considered overweight or obese while n = 641 (38%)) were classified as overweight or obese by IOTF standards (Table 1). In the latter group, a higher proportion of children was non-Hispanic black or lived in low-income households compared to the other population subgroups. Across all groups approximately 40% of the population was considered overweight or obese using the CDC cut-point of ≥85^th ^percentile on the age- and gender-specific BMI-for-Age growth chart.

**Table 1:** Description of the total population and three study subpopulations (in percent)

**Characteristics**	**Full sample of 2–18 year olds with dietary and medical data**	**Group a**	**Group b**	**Group c**
	**(n = 7,672)**	**(n = 3,467)**	**(n = 1,064)**	**(n = 641)**

**Number of children represented**	n = 63,642,060	n = 20,845,577	n = 7,681,593	n = 3,598,335
**Females**	49	48	50	43
**Ethnic group**				
**Non-Hispanic white**	61	61	66	54
**Non-Hispanic black**	14	15	13	19
**Mexican**	12	12	9	14
**Other**	12	12	12	12
**Household income**^c^				
**<1.3 PIR**	39	39	38	44
**1.3–1.84 PIR**	12	13	13	11
**1.85–3.4 PIR**	19	20	19	18
**>3.5 PIR**	29	28	30	28
**Weight status**^d^				
**Health-y weight**	62	61	62	59
**At risk for Overweight**	15	15	16	16
**Overweight**	23	24	22	25

^a ^Study populations:

Group a): Non-missing data: ≥ three criteria but missing blood glucose (2–18 year olds)

Group b) Non-missing data: ≥ three criteria & blood glucose, not overweight or obese^b ^(12–18 year olds)

Group c) Non-missing data: ≥ three criteria & blood glucose, not overweight or obese^b ^(12–18 year olds)

^b ^Using International Obesity Task Force (IOTF) standards,

^c ^Poverty Income Ratio (PIR),

^d ^CDC gender specific BMI-for-Age growth chart: healthy weight (6–84^th ^percentile), at risk for overweight (85–94^th ^percentile), overweight (≥ 95^th ^percentil)

### Prevalence of the Metabolic Syndrome

Examination of the proportion of children meeting the diagnostic criteria for MS showed that teenagers classified as overweight or obese had the highest prevalence of meeting any of the five criteria. Two percent of the children ages 2–18 years old with dietary and medical data but no blood glucose measurements met the diagnostic criteria for MS (meets or exceeds at least three of the fiver criteria). In the group of children with blood glucose measurements only 0.7% of the non-obese had MS compared to 23% in the overweight or obese 12–18 year olds (Table 2).

**Table 2:** Prevalence of risk factors for the Metabolic Syndrome (MS) in a nationally representative sample of children (in percent)

**Criteria**		**Group a**	**Group b**	**Group c**
		**(n = 3,467)**	**(n = 1,064)**	**(n = 641)**

Blood Triglycerides	≥ 110 mg/dL	8%	18%	36%
High Density Lipoprotein	≤ 40 mg/dL	20%	16%	39%
Abdominal Obesity	≥ 90^th ^%tile waist circumference^a^	18%	1%	41%
Hyperglycemia	Fasting Glucose ≥ 100^b ^mg/dL	N/A	10%	16%
Blood pressure Systolic Diastolic	≥ 90th %tile^a ^or ≥ 90th %tile^a^	6%	5%	12%
Metabolic Syndrome	Meets ≥ 3 of the 5 criteria	2%	0.7%	23%

^a ^for age, sex and height, ^b ^cut-point updated to reflect the most current recommendation [10], ^c ^using IOTF guidelines

### Pattern analysis

Pattern analysis indicated that most children in either subpopulation did not meet any of the five criteria (62% in the group of children with non-missing data on at least three of the five diagnostic criteria but no fasting blood glucose data and 12–18 year olds with blood glucose data who were not overweight or obese and 31% in the 12–18 year olds with blood glucose levels who were overweight or obese (Table 3). Due to the missing information on hyperglycemia, the 2–18 year olds with missing data on blood glucose levels were only coded as "0/1" for the remaining four criteria. In that group, the most common pattern was low HDL only (pattern 0100 in 11% of the population) followed by abdominal obesity only (pattern 0010 in 8%), and low HDL together with abdominal obesity (pattern 0110 in 5%).

**Table 3:** Patterns of Metabolic Syndrome diagnostic criteria (in percent)^a^

**Group a): Non-missing data on ≥ 3 criteria but missing blood glucose data (2–18 years old) (n = 3,467)**					
**Triglycerides**	**HDL**^b^	**Obesity**	**Hyperglycemia**	**HTN**^c^	**Prevalence (in %)**

0	0	0	N/A	0	**62.3**
0	0	0	N/A	1	**3.1**
0	0	1	N/A	0	**8.3**
0	0	1	N/A	1	**0.9**
0	1	0	N/A	0	**11.0**
0	1	0	N/A	1	**0.6**
0	1	1	N/A	0	**5.2**
0	1	1	N/A	1	**0.9**
1	0	0	N/A	0	**3.6**
1	0	0	N/A	1	**0.2**
1	0	1	N/A	0	**1.0**
1	0	1	N/A	1	**0.2**
1	1	0	N/A	0	**1.8**
1	1	1	N/A	0	**1.0**
1	1	1	N/A	1	**0.1**

**Group b): Non-missing data: ≥ 3 criteria & blood glucose but not overweight/obese (12–18 years old) (n = 1,064)**					

**Triglycerides**	**HDL**^b^	**Obesity**	**Hyperglycemia**	**HTN**^c^	**Prevalence (in %)**

0	0	0	0	0	**61.9**
0	0	0	0	1	**2.2**
0	0	0	1	0	**6.4**
0	0	1	0	0	**0.1**
0	1	0	0	0	**9.1**
0	1	0	0	1	**0.9**
0	1	0	1	0	**0.4**
0	1	0	1	1	**0.2**
1	0	0	0	0	**10.8**
1	0	0	0	1	**1.0**
1	0	0	1	0	**1.6**
**1**	**0**	**0**	**1**	**1**	**0.1**
**1**	**1**	**0**	**0**	**0**	**4.4**
**1**	**1**	**0**	**1**	**0**	**0.4**

**Group c): Non-missing data: ≥ 3 criteria & blood glucose & overweight or obese (12–18 years old) (n = 641)**					

**Triglycerides**	**HDL**^b^	**Obesity**	**Hyperglycemia**	**HTN**^c^	**Prevalence (in %)**

0	0	0	0	0	**30.6**
0	0	0	0	1	**2.4**
0	0	0	1	0	**3.4**
0	0	0	1	1	**0.2**
0	0	1	0	0	**10.7**
0	0	1	0	1	**1.1**
0	0	1	1	0	**1.4**
**0**	**0**	**1**	**1**	**1**	**0.7**
0	1	0	0	0	**6.2**
0	1	0	0	1	**0.8**
0	1	0	1	0	**0.9**
0	1	1	0	0	**4.2**
**0**	**1**	**1**	**0**	**1**	**1.0**
**0**	**1**	**1**	**1**	**0**	**0.7**
**0**	**1**	**1**	**1**	**1**	**0.2**
1	0	0	0	0	**3.5**
1	0	0	0	1	**0.3**
1	0	0	1	0	**1.3**
**1**	**0**	**0**	**1**	**1**	**0.1**
1	0	1	0	0	**3.4**
**1**	**0**	**1**	**0**	**1**	**1.1**
**1**	**0**	**1**	**1**	**0**	**0.5**
**1**	**0**	**1**	**1**	**1**	**0.7**
1	1	0	0	0	**7.3**
**1**	**1**	**0**	**0**	**1**	**0.9**
**1**	**1**	**0**	**1**	**0**	**1.3**
**1**	**1**	**1**	**0**	**0**	**11.2**
**1**	**1**	**1**	**0**	**1**	**0.7**
**1**	**1**	**1**	**1**	**0**	**1.7**
**1**	**1**	**1**	**1**	**1**	**2.0**

^a^1 = met the criteria, 0 = did not meet the criteria, ^b^High Density Lipoprotein, ^c^Hypertension

In the subpopulation of 12–18 year olds with blood glucose data who were not overweight or obese, the most common patterns were high triglycerides only (pattern 10000 in 11% of the population), followed by low HDL alone (pattern 01000 in 9%) and hyperglycemia alone in 6%). Overall, 8% of the children in this group met the criteria for hyperglycemia and 5% had dyslipidemia (met the criteria for high triglycerides together with how HDL).

In the subgroup of 12–18 year olds who provided blood glucose levels and were also classified as overweight or obese, 2% met all five diagnostic criteria (pattern 11111). The three most common patterns were dyslipidemia and abdominal obesity or abdominal obesity alone (pattern 11100 and pattern 00100 each in 11% of the population) and dyslipidemia alone (pattern 11000 in 7%). Overall, 41% of the children in this group met the criteria for hyperglycemia and 25% had dyslipidemia.

In the combined sample of children with blood glucose data, 11% of the children met the criterion for hyperglycemia.

## Discussion

Due to the rapidly increasing number of children with excessive body weight in the U.S., the proportion of children developing the Metabolic Syndrome is likely to increase as well. Similar to adults, prevention and treatment might be achieved with changes in children's eating behavior and other lifestyle factors [19,20]; however, there is lack of research indicating a relationship between MS and diet in children.

MS has been be assessed using varying diagnostic criteria in the pediatric population and there is consensus that the potential development of MS in children is reason for concern [21-23]. Nevertheless, there is an urgent need to establish one uniform set of diagnostic guidelines for the diagnosis of pediatric MS [24-26].

Based on the data in this nationally representative data set, it is apparent that large proportions of children are at high risk for developing the disease. Particularly the proportion of adolescents with dyslipidemia (concurrent elevated total triglycerides and low HDL levels) was high even in children who were not overweight or obese. Furthermore, in the sample including the younger children who did not provide fasting glucose levels, the proportion of children meeting at least three indicators of MS was less than one percent. Since that particular group could only meet four of the five diagnostic criteria, this estimate does not provide a fair comparison of disease risk. If one would assume a diagnostic criteria set of meeting at least two of the remaining four criteria (ignoring hyperglycemia as a risk factor), then 12% of the child population in this study would have been considered as having MS. Thus, even in young children and when only considering dyslipidemia (high triglycerides concurrent with low HDL), abdominal obesity, and hypertension, the risk for MS is approaching public health importance.

Not surprisingly, we found that in teenagers 12–18 years old who provided fasting blood glucose levels but were not classified as overweight or obese, fewer individuals met the criteria for MS compared to their overweight or obese peers. However, even in the non-overweight or obese group, the proportion of children meeting the criteria for dyslipidemia, hyperglycemia, or hypertension was very high.

Limitations of this study include that all laboratory and medical data in the NHANES data set are based on one measurement, thus, results reported here are based on the assumption that measured values are representative of the individual's averages levels for blood triglyceride levels, HDL, and fasting blood glucose. Due to missing data on fasting blood levels in children under 12 years old, the overall prevalence of the disease in the entire child population can not be accurately estimated and subpopulations needed to be created. Based on the nationally representative character of the data, this stratification was possible without the loss of statistical power.

The use of a waist circumference to determine abdominal obesity rather than using population-specific BMI cut points (as in the CDC BMI-for-age growth charts or the IOTF standards) leads to lower estimates of children at risk for disease. In this sample, only approximately half of the children considered overweight or obese using the BMI were also found to have abdominal obesity. However, due to the positive association between abdominal fat and risk factors of cardiovascular disease, some have proposed the use of waist circumference alone (rather than a combination of indicators for hypertension, dyslipidemia, and insulin resistance) for the diagnosis of MS [9].

As our data showed, the prevalence of the MS in U.S. children and adolescents is high and the patterns of children's risk factors vary. Overall, the pediatric population might benefit from increase public health messages on the importance of consuming a diet moderate in fat and higher in foods that promote high HDL levels. Especially children from socio-demographic and ethnic backgrounds that are at higher risk to become overweight [27] or develop the MS [28,29] might benefit from changes in dietary intake patterns. Public policy to prevent pediatric MS should not be limited to the achievement of healthy body weight but also encourage increased consumption of high quality diet to promote decreasing total triglyceride levels while increasing HDL levels.

## Conclusion

Our data shows that the proportion of children and adolescents at risk for the Metabolic Syndrome is high. Diet and life style changes might help prevent the development of the disease not only by preventing the onset of childhood obesity but also by improving diet quality to lower the risk for dyslipidemia in children independent of their weight status.

## List of Abbreviations

CDC = Centers for Disease Control and Prevention

HDL = High Density Lipoproteins

IOTF = International Obesity Task Force

MS = Metabolic Syndrome

NHANES = National Health and Nutrition Examination Survey

## Competing interests

The author(s) declare that they have no competing interests.

## Authors' contributions

SK conceived of the study, guided the statistical analysis and interpretation of results as well as led the manuscript preparation. LJM contributed to data analysis and interpretation of res-ults as well as the writing of the manuscript. DAW led the data management efforts and conducted data analysis. All authors contributed to the manuscript.
